# A Comprehensive Strategy for Stepwise Design of a Lab PROTOTYPE for the Removal of Emerging Contaminants in Water Using Cyclodextrin Polymers as Adsorbent Material

**DOI:** 10.3390/ijms25052829

**Published:** 2024-02-29

**Authors:** Antonio Tomás Hernández Cegarra, Teresa Gómez-Morte, José Antonio Pellicer, Nuria Vela, María Isabel Rodríguez-López, Estrella Núñez-Delicado, José Antonio Gabaldón

**Affiliations:** 1 Molecular Recognition and Encapsulation Research Group (REM), Health Sciences Department, Universidad Católica de Murcia (UCAM), Campus de los Jerónimos 135, E-30107 Guadalupe, Spain; athernandez@alu.ucam.edu (A.T.H.C.); tgomez@ucam.edu (T.G.-M.); japellicer@ucam.edu (J.A.P.); mirodriguez@ucam.edu (M.I.R.-L.); enunez@ucam.edu (E.N.-D.); 2 Applied Technology Group to Environmental Health, Universidad Católica de Murcia (UCAM), Campus de los Jerónimos 135, E-30107 Guadalupe, Spain; nvela@ucam.edu

**Keywords:** β-cyclodextrins, porous adsorbent, adsorption kinetics, pilot design, furosemide, hydrochlorothiazide

## Abstract

The significant environmental issue of water pollution caused by emerging contaminants underscores the imperative for developing novel cleanup methods that are efficient, economically viable, and that are intended to operate at high capacity and under continuous flows at the industrial scale. This study shows the results of the operational design to build a prototype for the retention at lab scale of pollutant residues in water by using as adsorbent material, insoluble polymers prepared by β-cyclodextrin and epichlorohydrin as a cross-linking agent. Laboratory in-batch tests were run to find out the adsorbent performances against furosemide and hydrochlorothiazide as pollutant models. The initial evaluation concerning the dosage of adsorbent, pH levels, agitation, and concentration of pharmaceutical pollutants enabled us to identify the optimal conditions for conducting the subsequent experiments. The adsorption kinetic and the mechanisms involved were evaluated revealing that the experimental data perfectly fit the pseudo second-order model, with the adsorption process being mainly governed by chemisorption. With K_F_ constant values of 0.044 (L/g) and 0.029 (L/g) for furosemide and hydrochlorothiazide, respectively, and the determination coefficient (R^2^) being higher than 0.9 for both compounds, Freundlich yielded the most favorable outcomes, suggesting that the adsorption process occurs on heterogeneous surfaces involving both chemisorption and physisorption processes. The maximum monolayer adsorption capacity (q_max_) obtained by the Langmuir isotherm revealed a saturation of the β-CDs-EPI polymer surface 1.45 times higher for furosemide (q_max_ = 1.282 mg/g) than hydrochlorothiazide (q_max_ = 0.844 mg/g). Based on these results, the sizing design and building of a lab-scale model were carried out, which in turn will be used later to evaluate its performance working in continuous flow in a real scenario.

## 1. Introduction

Global water scarcity is now a widespread issue impacting every continent, posing a significant challenge in today’s world. Over the past century, the consumption and depletion of water resources have surged at twice the rate of population growth. Despite an overall abundance of drinking water on Earth, it is unevenly distributed, subject to wastage and pollution in certain regions, and frequently lacks sustainable management [1,2].

The distribution and quality of water in Europe have raised significant concerns, leading member states to initiate efforts for water conservation and improved water management. The European Union has instituted a comprehensive framework with the goal of actively preventing and efficiently managing water pollution. This framework includes various measures designed to assess the chemical condition of water and reduce the presence of pollutants [3]. Directive 2013/39/EU, an amendment to Directives 2000/60/EC and 2008/105/EC pertaining to priority substances within the domain of water policy [4], places considerable importance on pinpointing the underlying causes of pollution. It advocates for the adoption of environmentally sustainable approaches to treat and curtail pollutant emissions at their source. Additionally, the Sustainable Development Goals (SDGs) have incorporated several strategies initially outlined in the Millennium Development Goals (MDGs), with a specific focus on Goal 6, which strives to “Ensure availability and sustainable management of water and sanitation for all” [5].

Despite the implementation of significant pollution control measures throughout the past century, which have successfully led to substantial reductions in various pollutants, such as persistent organic pollutants, a troubling trend has surfaced. There is a noticeable increase in the prevalence of emerging contaminants (ECs), presenting potential risks to both the environment and human health [6]. These contaminants encompass a variety of natural or synthetic chemical substances originating from diverse sources, including pharmaceuticals (PhACs), personal care products, drugs, preservatives, plasticizers, and pesticides, among others [7,8].

Urban wastewater is a major source of water pollution when it is not properly collected and treated [8,9]. Consequently, in recent years, developed countries have tightened regulations governing effluents and their permissible composition for release into the environment. The current European Directive sets minimum requirements for the collection, treatment, and discharge of urban wastewater [10]. This directive has proven to be very effective in reducing water pollution and improving the treatment of wastewater discharge over the last three decades, but the European authorities are reviewing this standard in order to align it with the objectives of the European Green Pact. The revision of Directive 91/271 aims to improve the protection of Europeans’ health and the environment by addressing issues such as pollution in smaller cities, stormwater overflows, micropollutants, climate change, energy efficiency, and resource management. It also seeks to ensure access to basic sanitation for all EU citizens, especially for the most vulnerable and marginalized groups.

Conventional technologies employed in wastewater treatment plants (WWTPs) have proven ineffective in removing ECs [8,11]. Current WWTPs were not specifically developed for the elimination of ECs, the traditional methods used, such as coagulation/flocculation, precipitation, biodegradation, filtration, or carbon filters, are not capable of successfully removing ECs or have the disadvantage of being expensive and having a high cost of regeneration as is the case of adsorption with activated carbon [11,12]. As a result, various studies are exploring alternatives to eliminate ECs while remaining environmentally and economically sustainable [13,14,15].

Cyclodextrins (CDs), cyclic oligosaccharides derived from the enzymatic breakdown of starch, have been extensively researched in the past century because of their capability to form inclusion complexes [16,17]. CDs have a torus-shaped structure, with the internal cavity being hydrophobic and the external cavity being hydrophilic. Inside the internal cavity, CDs are capable of hosting numerous molecules of an organic or inorganic nature, forming the so-called inclusion complexes [18,19]. The formation of these inclusion complexes with numerous ECs has been and is currently one of the most important applications of CDs, they are part of numerous formulations available on the market [16,18]. Consequently, CDs have been considered a potential solution for enhancing water quality [20,21]. 

However, their water-soluble nature needs chemical synthesis methods including cross-linking, immobilization on a solid support such as inorganic or organic fibers or magnetic particles, and self-assembly, to render them suitable for treating contaminated water. Between them, cross-linked cyclodextrin polymers have been widespread used due to their simplest preparation method via different crosslinking agents such as epichlorohydrin (EPI) [22], EDTA [23], citric acid [24], glutaraldehyde [25], tetrafluoroterephthalonitrile [26], or 1-4-diazabicyclooctane-1,4-butanediol diglycidyl ether [27] and excellent adsorption performances regarding traditional sorbents such as activated carbon or ion exchange resins.

In this sense, water-insoluble CD polymers have emerged as a crucial option for removing pollutants from wastewater, that once saturated, could be regenerated using a chaotropic agent, allowing them to be reused in several adsorption cycles. In this sense, numerous efforts have been carried out to synthesize cyclodextrin polymers with the goal of enhancing both their adsorption capacity and versatility, being able to form inclusion complexes with a broad range of target molecules, since the polymer acquires amphiphilic characteristics through the formation of a three-dimensional network of greater extent. This results in both hydrophilic and hydrophobic properties attributed to the presence of glucose units (CDs) and primarily hydroxyl groups and, the methyl groups and ether bonds of the cross-linking agent, as well as CD-glyceryl bonds, respectively. Therefore, they have been widely applied for environmental purposes, in line with the One Health European strategy, particularly in the removal of heavy metals by complexation, electrostatic interactions, ion exchange, or via covalent bonding to external hydroxyl groups that provide an acidic media [28,29,30]; and other EPs recurrent in aqueous media such as dyes [31,32,33], pesticides [34,35,36] and other micropollutants [26,37,38]. Despite how successful the adsorption of ECs on CD polymers is, not many pilot-scale demonstrations have been published [38].

CDs and EPI polymers have been widely studied in the last decade due to their relative ease of synthesis and the excellent results obtained for the adsorption of different molecules in wastewater treatment and other applications such as biomedicine [39]. This highlights the importance of not only considering the characteristics of the polymeric material (such as CD content, degree of cross-linking, swelling ratio, and pore size), but also understanding the interactions among the three components of the sorption system, the polymer, the pollutant, and the water to be cleaned, to achieve optimal results. Nonetheless, diverse physical and chemical mechanisms come into play between the polymeric sorbent and the targeted pollutant to be removed. Primarily, a chemisorption mechanism occurs by inclusion into the cyclodextrins (CDs) cavity. Additionally, there is the potential to sequester pollutants through cooperative effects between CDs and/or via additional interactions within the mesh, facilitating diffusion into the polymer network [40]. Furthermore, the complexity of interpretations may be heightened by considering the structure and polarity of the pollutants, along with the experimental conditions of the batch (such as material dosage, pollutant concentration, pH, ionic strength, etc.). This challenge could become more pronounced when studying real wastewater containing multiple contaminants.

The present manuscript examines, by in-batch assays, the performances of a β-CDs-EPI polymer to obtain the required parametric values for the correct design of a prototype that could operate under a real scenario at continuous flows. For that, the adsorption phenomena on the β-CDs-EPI polymer of two diuretics drugs that are widely used and recurrent in wastewater, such as furosemide and hydrochlorothiazide, were evaluated as model pollutants. Experimental data obtained in batch assays were adjusted to different kinetics models and adsorption isotherms to understand the physic-chemical characteristics of this adsorbent material for pollutant removal. The results obtained were used as input parameters for the theoretical stepwise design of the flow-through prototype, starting with the establishment of basic processes of adsorption/desorption mechanisms, including work ranges and ratios; definition and elaboration of a flowchart to identify each of the stages and equipment involved; selection of the column diameter based on satisfactory volume of adsorbent for continuous work, conditioning the design of the rest of components of the prototype; and definition of process steps (adsorption, regeneration, rinsing).

## 2. Results and Discussion

The adsorption process of furosemide and hydrochlorothiazide on β-CDs-EPI polymer was explored as a function of some key experimental parameters including the quantity of adsorbent, pH impact, agitation speed, temperature, and diuretic concentration, as a crucial step for defining the requirements of a laboratory prototype aimed at efficient EC removal from water. A prerequisite for upscaling the process is to ensure a rapid and effective adsorption capacity. If the duration needed to attain adsorption equilibrium between the β-CDs-EPI adsorbent and wastewater is excessively long, in-flux operational feasibility is compromised, limiting the potential applications in real-world scenarios. Following a comprehensive assessment of these factors, as previously described by our research group working with the same polymer [41,42], the experimental findings revealed that using 1 g of polymer at pH 7.0 working in a reaction volume of 50 mL at room temperature, with an agitation speed of 500 rpm yielded the most favorable conditions for conducting subsequent experiments.

### 2.1. Effect Contact Time

**Figure 1:**
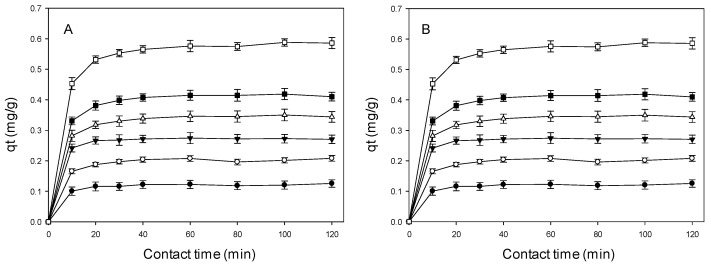
The effect of the time of contact on adsorption capacity of β-CDs-EPI polymer at different concentrations of pharmaceuticals. (**A**) Furosemide 5.0 mg/L (●), 7.5 mg/L (○), 10 mg/L (▼), 12.5 mg/L (Δ), 15 mg/L (■), 20 mg/L (□). (**B**) Hydrochlorothiazide 5.0 mg/L (●), 7.5 mg/L (○), 10 mg/L (▼), 12.5 mg/L (Δ), 15 mg/L (■), 20 mg/L (□). N = 3.

Figure 1A,B showed the adsorption data of the β-CDs-EPI polymer against different concentrations of the diuretics studied (between 5 and 20 mg/L), as a function of the time of contact. The analysis of the results shows that in all the tested concentrations, the adsorption of the pollutant by the polymer increases until reaching a constant value in a determined time. At that point, the polymer is in dynamic equilibrium between the amount of PhACs adsorbed and the amount of PhACs desorbed from the polymer, the equilibrium time being the interval required for the uptake of pollutants from the solution by the polymer to stop. The amount of PhACs that are adsorbed up to the equilibrium time is the maximum adsorption capacity of the polymer under the conditions in which the experiment was accomplished [41].

Several stages can be identified in the furosemide adsorption process, in the first 10 min of contact time, very rapid adsorption occurs, and between 10 and 20 min of the adsorption process, equilibrium is reached. However, as can be seen in Figure 1B, for hydrochlorothiazide the equilibrium time was reached at 60 min of contact time, except for 5 mg/L where the maximum adsorption capacity occurred at 40 min.

Once equilibrium is established, there is a balance between the adsorption and desorption of pollutants within and outside the polymer. The interval required to achieve this equilibrium is denoted as the time at equilibrium, and the quantity of pollutant retained by the polymer specifies its maximum absorption capacity.

### 2.2. Kinetic Analysis

To clarify the adsorption kinetic process and the involved mechanisms, the experimental data underwent analysis using various kinetic models: pseudo-first-order, pseudo-second-order, and intraparticle diffusion (see Table S1 Supplementary Materials). The determination coefficient obtained in the adjustment (R^2^) was the key to understanding the mechanism underlying the adsorption process.

For furosemide, the R^2^ values obtained for the pseudo-first-order model are in a range of 0.987 to 0.834 for β-CDs-EPI. In the case of hydrochlorothiazide, the R^2^ values are in a similar range (0.991 to 0.805).

The values of experimental *qe* and calculated *qe* were different, indicating that the adsorption process does not fit the pseudo-first-order model (see Supplementary Materials Figure S1A,B), these results are in agreement with those published for other PhACs with CDs polymers such as the case of ciprofloxacin and different organic pollutants where the pseudo first order model did not show a suitable adjustment to the experimental data [27,43,44]; therefore, the pseudo second order model was applied. 

By fitting experimental data to the pseudo-second-order model, researchers can predict how fast pollutants will be removed from a solution under different conditions, such as varying concentrations or temperatures. This predictive capability is crucial for optimizing adsorption processes and designing efficient treatment systems.

The results of the graphical representation (see Supplementary Materials Figure S1C,D) of t/qt versus contact time show a straight line for the two PhACs studied and the polymer used. The fit values (R^2^), all higher than 0.99, reveal that the experimental data perfectly fit this kinetic model. Furthermore, the values of experimental *qe* and *qe* calculated are similar (see Table S1 Supplementary Materials). The adjustment to the PSOM model indicates that this model assumes that adsorption occurs through chemisorption, involving the formation of chemical bonds between the adsorbate and the adsorbent surface. These results are in agreement with those published for other pharmaceuticals such as ciprofloxacin and different dyes [27].

Different stages are involved in the adsorption process, such as the transport of pharmaceutical molecules to the surface of the adsorbent material and the subsequent diffusion of PhAC molecules into the polymer. In addition, it was studied whether intraparticle diffusion is the process that determines the rate of adsorption. The intraparticle diffusion model helps in understanding the mechanism of mass transfer within the porous structure of the adsorbent material. It describes the rate-limiting step associated with the diffusion of adsorbate molecules from the bulk solution into the internal pores of the adsorbent.

From the graphical representation of the model (see Supplementary Materials Figure S1E), two zones can be distinguished for furosemide, the first one being more curved and the later stable zone corresponding to an intraparticle diffusion. On the other hand, for hydrochlorothiazide, a linear rise was observed at the beginning and later a plateau (see Supplementary Materials Figure S1F), especially at the highest concentrations (15 and 20 mg/L). 

The plateau stage means that intraparticle diffusion begins to decrease because there is not enough pollutant available. As can be seen in Table S1 (see Supplementary Materials), the value of *ki* increases with the concentration. These results show that intraparticle diffusion is involved in the adsorption processes, but that it is not the only mechanism employed, there being other mechanisms such as the formation of inclusion complexes, adsorption on the external surface of the polymer, ionic exchange, and the diffusion into the polymeric network [43]. The amphiphilic cross-linked EPI-β-CD polymer exhibits both hydrophobic and hydrophilic cavities with a chaotic nature, resulting in a random distribution of shapes and sizes. This aspect is crucial for interpreting the obtained results. The hydrophilic properties of the polymer facilitate interaction with water, enhancing hydration through the potential formation of hydrogen bonds. Additionally, the polymer’s hydrophilicity promotes better swelling of the network, thereby increasing its potential for diffusion. Furthermore, the hydrophobic cavities of the CDs and the cage structures formed by the cross-linker, abundant in ethylene oxide groups, attract hydrophobic PhACs [37].

### 2.3. Adsorption Equilibrium

The distribution in the equilibrium of the PhACs between the polymer and the solution was studied, and this was carried out by applying the Freundlich, Langmuir, and Tempkin isotherm models [45]. Table 1 shows the parametric values obtained for each model.

**Figure 2:**
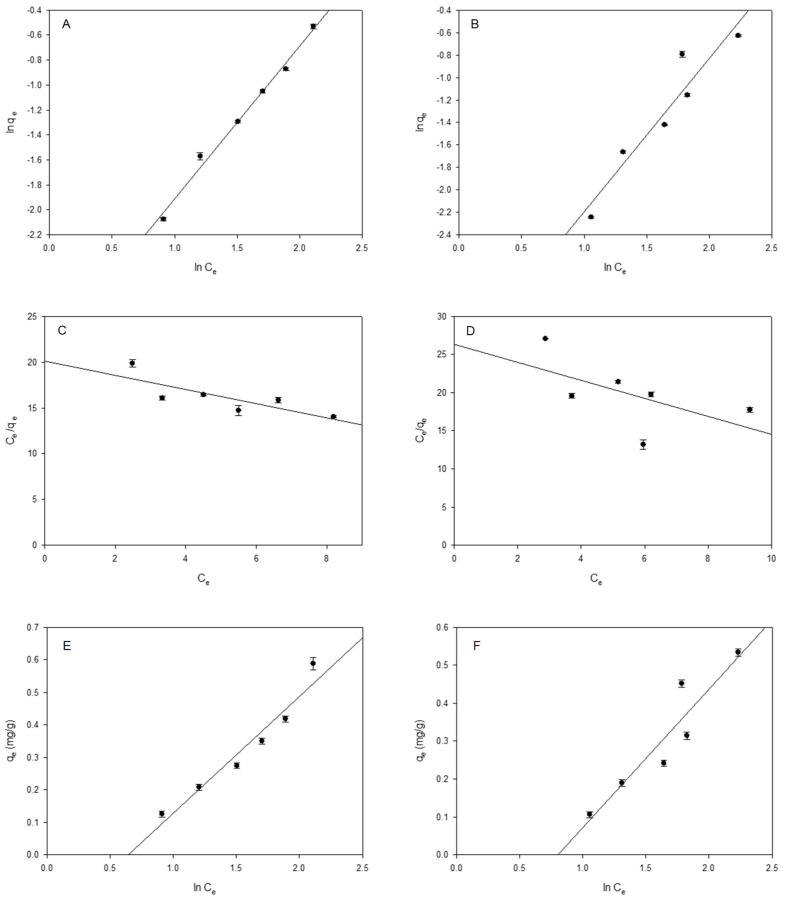
Isotherm analysis (**A**,**B**) Freundlich model; (**C**,**D**) Langmuir model; (**E**,**F**) Tempkin model for Furosemide (**A**,**C**,**E**) and Hydrochlorothiazide (**B**,**D**,**F**) by β−CDs−EPI polymer. N = 3. Black circles agree with the different concentrations of PhACs tested: 5.0 mg/L, 7.5 mg/L, 10 mg/L, 12.5 mg/L, 15 mg/L and, 20 mg/L.

The Freundlich isotherm is particularly useful for describing non-ideal adsorption behavior, where the adsorption capacity of the surface varies with the concentration of the solute in the solution. Unlike the Langmuir isotherm, which assumes monolayer adsorption on a homogeneous surface, the Freundlich isotherm allows for multilayer adsorption on heterogeneous surfaces, a linear representation was obtained for the two diuretics studied (Figure 2), reaching K_F_ constants values of 0.044 (L/g) for furosemide and 0.029 (L/g) for hydrochlorothiazide. The order of K_F_ values suggests that furosemide exhibits the strongest adsorption and the highest capacity at the β-CDs-EPI polymer surface, followed by hydrochlorothiazide. In addition, the determination coefficients (R^2^) of 0.991 and 0.905 for furosemide and hydrochlorothiazide, respectively (Table 1), indicated that the Freundlich equation fitted the adsorption data better than the Langmuir and Temkin models. 

The magnitude of the Freundlich exponent n_F_ for hydrochlorothiazide and furosemide, which ranged from 0.737 to 0.817, respectively, indicates that the sorption mechanism is controlled by adsorption and not absorption in the Freundlich model, giving a rational description of the experimental data, involving both chemisorption and physisorption processes, agreeing with other published studies [27,46]. The role of the Freundlich isotherm in adsorption lies in its ability to describe multilayer adsorption onto heterogeneous surfaces. It is particularly useful when the adsorption process does not follow straightforward monolayer adsorption (as assumed in models such as the Langmuir isotherm) and when multiple layers of solute can be adsorbed onto the surface with varying energies. Moreover, the exponent highlights the variety of energies linked to the adsorption of both diuretics on the β-CDs-EPI polymer surface. Moreover, n < 1 for furosemide and hydrochlorothiazide indicates that, upon increasing the PhACs concentration/loading, the binding energy between the surfaces and both compounds is reduced. 

The Langmuir model assumes that adsorption occurs on a homogeneous surface where adsorbate molecules form a monolayer. This means that each adsorption site on the surface can only be occupied by one molecule, and no further adsorption can occur once the surface is fully covered. The isotherm postulated that all sites showed uniform surface coverage [47]. Unlike the Freundlich isotherm, the values of determination coefficients (R^2^) (Table 1) for the Langmuir model were lower in all cases, which shows that the data do not fit well with this isotherm. 

The most important parameter obtained with the Langmuir model is the maximum monolayer adsorption capacity (q_max_), under the studied conditions, being 1.282 mg/g for furosemide and 0.844 mg/g for hydrochlorothiazide, showing that the order of saturation of the β-CDs-EPI polymer surface with diuretic, per mg diuretic, was 1.45 times higher for furosemide. This trend fits well with K_F_ values previously described by the Freundlich model. 

In addition, with the Langmuir isotherm, it was analyzed whether the adsorption process is favorable or not, with the separation factor (R_L_). The adsorption process is considered favorable when it is between 0 and 1. In the case, of furosemide and hydrochlorothiazide, in the concentration studies, the adsorption process on β-CDs-EPI polymer is favorable.

The Langmuir parameter K_L_ increased in the order 0.038 L/g (hydrochlorothiazide) <0.050 L/g (furosemide). This constant is mainly related to the adsorption energy and could give information about the β-CDs-EPI polymer–PhAC interaction and binding process strength. A higher K_L_ value indicates stronger adsorption affinity.

Thus, based on the Langmuir model, one expects that the β-CDs-EPI polymer–PhAC interaction increases in the order hydrochlorothiazide < furosemide. The same order but with different values 0.448 L/g (hydrochlorothiazide) <0.525 L/g (furosemide), was obtained with the Temkin binding constant a_T_, which is also related to the binding strength. 

Lastly, the equilibrium experimental data were analyzed by fitting the results to the Tempkin isotherm (Figure 2). It assumes the linear decrease in the heat of adsorption of all the molecules found in the external layer to be a consequence of the interactions that occur between the polymer and the PhACs, in this case, the binding energies are uniformly distributed [48].

For ionic exchange, the binding energies are between 8 and 16 kJ/mol, and for physisorption they are between −40 kJ/mol. The Temkin b_T_ values for furosemide (6.890 kJ/mol) and hydrochlorothiazide (6.79 kJ/mol), suggest that the heat of adsorption on the β-CDs-EPI polymer increases in the order hydrochlorothiazide < furosemide as well as that physical and chemical processes are involved in the adsorption. Other than the Temkin constant b_T_, all the parameters of the represented isotherm models in Figure 2, refer to stronger adsorption and higher capacity for the furosemide diuretic compared to hydrochlorothiazide by considering the number of PhAC mg. 

### 2.4. Thermodynamic Parameters

The spontaneity of chemical reactions is related to the value of the Gibbs free energy (Δ*G*°). Prior to this calculation, it is necessary to determine the value of *K*° according to the Equation (1):

(1) $$K{}^{\circ}=K_{p}*M_{adsorbate}*\;55.5$$

where *K_p_* is the equilibrium constant (L/g), *M_adsorbate_* is the molecular weight of furosemide or hydrochlorothiazide, and 55.5 is the constant related to the mole concentration of water (mol/L) [49,50]. The results obtained in the previous Equation (1) were then used to calculate the spontaneity of the adsorption process following Equation (2).

(2) ∆G°=−RTlnln⁡K°

At room temperature, the standard free energy (Δ*G*°) for the β-CDs-EPI polymer was determined to be −16,919.810 J/mol for furosemide and −16,730.651 J/mol for hydrochlorothiazide, thus negative Δ*G*° values obtained at 25 °C demonstrated the inherent spontaneity of the adsorption process. It is also worth noting that a negative Δ*G*° indicates that the reaction is thermodynamically favorable in the forward direction. However, it does not provide information about the rate of the reaction or whether the reverse reaction is also spontaneous.

### 2.5. Polymer Reusability

The β-CD-EPI polymer could be reused for at least 10 cycles without losing capability significantly (>90%), which allows the reuse of both the PhACs and water, in a new adsorption cycle. Additionally, the polymer can be recycled for a subsequent round of pharmaceutical removal, aligning with the principles of the circular economy. 

### 2.6. Design Continuous Flow Prototype Adsorption

Once the batch adsorption data of the β-CDs-EPI polymer using two PhACs as pollutant models had been determined in distilled water, and the adsorption mechanism established by applying kinetic (pseudo-first-order, pseudo-second-order, and intraparticle diffusion) and equilibrium (Freundlich, Langmuir, and Tempkin isotherm) models, where both physical and chemical processes are involved, a laboratory-scale prototype was designed to validate in subsequent tests its behavior in real scenarios working continuously.

For that, a stepwise approach was followed starting with, (i) the characteristics of the pollutant solution and the adsorbent (synthesized as described in Section 3.2). In our case, we ascertained the adsorbent performances in batch assays of β-CDs-EPI polymer (Table 2), working solutions of furosemide, and hydrochlorothiazide in distilled water at concentrations between 5 and 20 mg/L; (ii) the establishment of basic processes of adsorption/desorption mechanisms, including work ranges and ratios. As in any engineering process, these two points are key to obtaining good execution and results of the engineering project. 

In this sense, we confront a substantial challenge since we must design and build equipment that works in continuous flow in a way that allows us to confirm the results obtained in the laboratory, and also provide sufficient information to validate in a future the operation of the process on an industrial scale.

Once these two points are stated, we must define the adsorption and desorption process steps, as well as the equipment necessary for their accomplishment. To do this, as a starting point, we must prepare a summary (Table 3) with the recommended design parameters for a standard adsorption system.

As a result of these input parameters, the next step is (iii) the definition of the stages of the process, together with the elaboration of a flowchart that helps us identify and understand each of the stages and equipment involved.

Thus, to get started with the design we selected an acceptable adsorbent volume for continuous work in the range of 1 to 3 L. After that, we carry out the necessary calculations to define the necessary column for the piloting. The selection of the column is the point of utmost importance in the design, conditioning its configuration and the design of the rest of the prototype components.

For that, adsorbent volumes of 1 L, 2 L, and 3 L were used to determine the necessary height and diameter of the column for the continuous process setting, for each adsorbent filling volume, a loading flow rates relation of 1:8 and 1:40 (i.e., 1 L adsorbent volume:8 L/h flow; 1 L adsorbent volume:40 L/h flow; 2 L adsorbent volume:16 L/h flow; 2 L adsorbent volume:80 L/h flow; 3 L adsorbent volume:24 L/h flow; 2 L adsorbent volume:120 L/h flow), and contact times of 7.5 min for 1:8 and 1.5 min for 1:40 rates. A column of Ø 90 mm capable of accommodating the β-CDs-EPI adsorbent polymer volumes, chosen with a final adsorbent bed depth not exceeding 550 mm nor 1200 mm for backwash expansion column height (the maximum recommended height), was selected. 

Transparent polyvinyl chloride with a design pressure of 6 bar was selected as the construction material of the column. This material has chemical compatibility with the PhACs and the desorbing (220 mM acetate buffer pH 4.0) solutions proposed for the pilot tests while allowing us to visualize the behavior of the process at different adsorption/desorption stages.

The columns shall be equipped with two polypropylene nozzles at the top and bottom that will retain the adsorbent within the column. The light passage is set at 100 μm, measured below the particle size of the adsorbent used. The loading and emptying operations of the adsorbent will be carried out through a removable accessory located at both ends of the column. 

Taking into account all the design criteria established above, we proceeded to determine the parametric values of the prototype for a column of 90 mm diameter (Ø) and one of 63 mm diameter (Table 4).

Taking into account the stated criteria of 1200 mm for the maximum recommended height in the expansion backwash process and 550 mm of bed depth column, we select Ø90 mm columns because the height for a smaller diameter column is Ø63 mm, exceeding the necessary heights of 350 mm for 2 L of adsorbent volume and flow rates of 16 L/h or 80 L/h, and 1130 mm for 3 L of adsorbent volume and flow rates of 24 L/h or 120 L/h.

Once the column was selected we proceeded with the definition of process stages, (iv) the adsorption step. As defined in the design parameters, the flow rates established for the adsorption process are in the range from 8 to 120 L/h, ensuring that the contact time between the emergent pollutant solution and the adsorbent β-CDs-EPI polymer will be within the values established in the stated design parameters (1 to 7.5 min).

The adsorption stage begins with the preparation of the PhACs solution in a 50 L tank TK-01 (as described in Section 3.8). This solution is sent to the adsorption column through a self-priming diaphragm pump (P-01), responsible for propelling the solution toward the column at the determined flow rate and pressure (2 to 4 bar). This flow rate is measured by an in-line flow meter FI-01 installed in the feed pipe, while the pressure is measured in the inlet and pressure gauges output of column at PI-01 and PI-02, respectively. To regulate the flow, a needle valve RG-01 is used. At the exit of the adsorption column, the treated PhAC solution will be collected in the product tank TK-02 (50 L). 

Next, it is necessary to estimate the (v) adsorption cycle. Based on the capacity data obtained from the adsorption isotherms in batch, and once stated, the volumes to be used in the columns, we carried out the necessary calculations to estimate the depletion of the β-CDs-EPI polymer used as adsorbent. These theoretical values are based on the data obtained from in-batch absorption processes with continuous stirring, but they provide us with the necessary information for a preliminary calculation of the pilot plant design (Table 5).

Now, we must propose in the design the two possible flow operation scenarios, co-current and counter-current (as described in Section 3.8). 

To operate co-current, the flow direction is downward. The PhAC solution enters through the upper part of the column, while the effluent exits through the lower part of the column through a polypropylene nozzle with a passage light of 100 µm. 

On the other hand, to operate counter-current, the flow direction is upward. The PhAC solution exits through the upper part of the column through a polypropylene nozzle (passage light of 100 µm) located at the upper part of the column. Tables S2 and S3 of Supplementary Materials detail the on/off positions of the valves when working in co-current (Table S2) and counter-current (Table S3) way, respectively.

As the prototype is designed to function continuously, the adsorbent polymer must undergo proper regeneration upon reaching its capacity limit by a backwash process. 

This involves the introduction of a desorbing solution to sponge the adsorbent restoring the capacity of the β-CDs-EPI polymer, ensuring complete contact of the solution with the adsorbent when the operating flow rate is between 55 and 110 L/h. The on/off positions of the valves to carry out the backwash process are the same as described above when working in counter-current flow (see Table S3 Supplementary Materials).

The (vi) desorption step starts when the β-CDs-EPI polymer loses its adsorbent capacity passing through a desorbing solution (as described in Section 3.8), to restore its adsorption capacity for the removal of PhACs. This solution is introduced in a 50 L tank TK-03, and the valves HV are placed in the desorption position (see Table S4 Supplementary Materials), turning on the pump P02. The desorbing solution flow rate (2 to 5 BV/h) is regulated with the RG-02 valve and the FI-01 flowmeter, while the pressure is measured in the inlet and pressure gauges output of column at PI-01 and PI-02, respectively. The contact time between the desorbing solution and the adsorbent β-CDs-EPI polymer will be within 20 to 60 min with the displacement of the desorbing solution being between 2 to 4 BV of water. At the exit of the desorption column, the desorbing solution will be returned to the tank TK-04 (50 L).

Finally, a rinsing process is carried out in order to remove possible traces of desorbing solution that remain in the column before starting a new adsorption process. The volume of effluent used is between 2 and 10 times higher than the volume of the β-CDs-EPI adsorbent polymer filled in the column. The on/off positions of the valves to carry out the rinsing process are the same as described above when working in co-current flow (see Table S2 Supplementary Materials).

**Figure 3:**
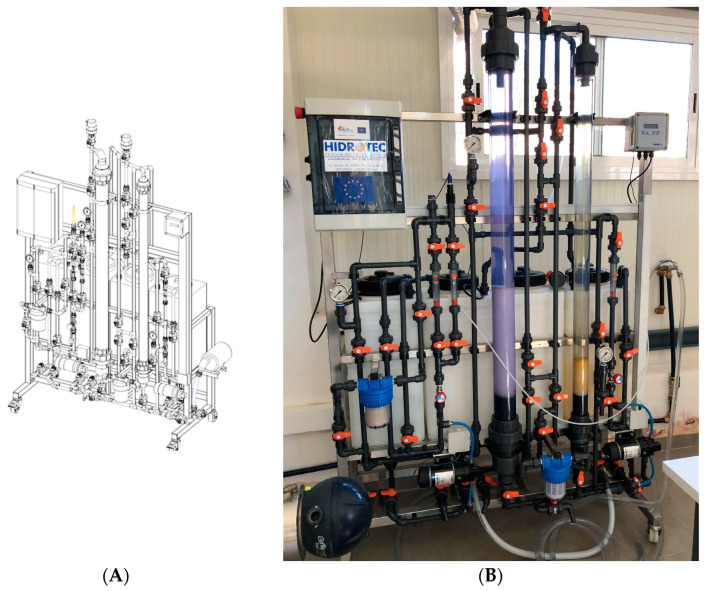
Image of the designed pilot-plant laboratory scale prototype (PPLSP). (**A**): Preliminary prototype drawing; (**B**): Full scale image of the built prototype.

Taking into account the theoretical calculations obtained using previous inputs, a pilot-plant laboratory scale prototype (PPLSP) was built to validate under continuous flows in subsequent tests (Figure 3), if fulfilled, the design criteria regarding the parameters obtained from the in-batch adsorption process.

As can be seen in Figure 3, we decided to include, in addition to the selected one (Ø90 mm), an additional smaller diameter column Ø63 mm not exceeding the necessary 1200 mm height (but lower bed depth working), in order to obtain comparative data of the adsorption process in a continuous way, as a function of the column size (see Supplementary Materials Table S5). 

The theory results of pharmaceutic retention using this prototype suggest that this simple and inexpensive technological setup could be scaled up to a functional field application to effectively capture emerging pollutants. To confirm this theoretical postulate, the built prototype should be subjected to representative continuous assays with water and wastewater samples enriched with emerging contaminants and the obtained results will be displayed in a succeeding work.

## 3. Materials and Methods 

### 3.1. Chemicals and Reagents

The β-cyclodextrins (β-CDs, CID 444041) used for the synthesis of the polymer were supplied by Arachem (Tilburgo, The Netherlands). The rest of the chemicals: epichlorohydrin (99%), sodium borohydride (98%), sodium hydroxide (98%), and acetone were from Sigma-Aldrich (Barcelona, Spain). The PhAC standards of Furosemide (CAS No.: 54-31-9, 100% purity) and Hydrochlorothiazide (CAS No.: 58-93-5, 100% purity) were supplied by Sigma-Aldrich (Barcelona, Spain).

### 3.2. Epichlorohydrin-β-Cyclodextrin Polymer Preparation

The β-CD-EPI polymer was synthesized following the method described by Pellicer et al. 2018 [32]. Firstly, 60 mg of sodium borohydride was mixed with 24 g of β-CDs and 24 mL of water at 50 °C. After 10 min of stirring, 26 mL of sodium hydroxide was added and the mixture was stirred for 5 min. Subsequently, 264 g of EPI was added dropwise. The mixture was kept under constant stirring for 6 h until the polymer was obtained. The adsorbent was washed with acetone and dried at 60 °C overnight. This work does not include the characterization of the β-CDs-EPI polymer since it was already published by this research group [41].

### 3.3. Diuretics Solution Preparation

To accomplish the adsorption experiments in batch, standard solutions of furosemide and hydrochlorothiazide, with molecular weights of 330.74 g/mol and 297.7 g/mol, respectively, were prepared daily at several concentrations (5.0; 7.5; 10.0; 12.5; 15.0, and 20.0 mg/L) in distilled water and used to enrich water aliquots at the described concentrations of each compound. After treatment with the polymer, the remaining PhAC concentration was measured in the supernatant using a spectrophotometer (Shimadzu UV-1603, Schimadzu Europe GmbH, Duisburg, Germany). Absorbance signatures were monitored upon treatment at the maximum absorbance of each compound (λ_max_ = 243 nm for furosemide; λ_max_ = 273 nm for hydrochlorothiazide) from the corresponding absorption spectra included in Supplementary Materials (see Figures S2 and S3).

### 3.4. Adsorption Experiments

Adsorption tests were carried out at room temperature (25 °C), using solutions containing different concentrations of PhACs ranging from 5 to 20 mg/L. In each assay test, a combination of 1 g of β-CDs-EPI polymer and 50 mL of PhAC solution was thoroughly mixed. After that, the mixture was stirred at 500 rpm. The amount of pollutant that remained unadsorbed by the polymer was determined at 10 min intervals (up to 40 min) and at 20 min intervals from 40 min to 120 min. The supernatant of the mixture was measured, after being subject to centrifugation (3000 rpm) for 5 min. Subsequently, the PhAC concentration was determined. Notably, all experiments were conducted in sets of three replicates. The measure of PhACs captured by the polymer (qe) was calculated using the following Equation (3) [51]:

(3) $$q_{e}=\frac{V(C_{0}-C_{e})}{m}$$

where V is the volume of PhACs solution (L), m is the mass of the employed polymer (g), *C*_0_ represents the initial concentration of PhACs in the liquid phase (mg/L), and *C*_e_ indicates the equilibrium concentration of PhACs in the liquid phase (mg/L). All experiments were conducted in triplicate.

### 3.5. Kinetics Analysis

With the aim of examining the PhAC adsorption mechanisms onto β-CDs-EPI polymer, three kinetics models were explored to assess the adsorption processes. These models include the pseudo-first-order kinetic model [52], the pseudo-second-order kinetic model [53,54], and the intraparticle diffusion model [55], which were evaluated using Equations (4)–(6), respectively.

(4) $$\log(q_{e}\;-\;q_{t})=logq_{e}-\frac{k_{1}}{2.303}t$$

(5) $$\frac{t}{q_{t}}=\frac{1}{k2qe^{2}}+\frac{1}{q_{e}}\;t$$

(6) $$q_{t}=ki\sqrt{t}+C$$

where *q_e_* and *q_t_* represent the quantity of adsorbed PhACs (mg/g) at equilibrium and at time *t* (min), respectively; *k*_1_ indicates the pseudo-first-order rate constant (min^−^^1^), *k*_2_ represents the equilibrium rate constant of pseudo-second order (g/mg min), *k_i_* the intraparticle diffusion rate constant (mg/g min^1/2^), *t* is the time and *C* is the intercept (mg/g).

### 3.6. Isotherms Analysis

For the optimization of the adsorption process of pollutants in adsorbent materials such as polymers, the study of their interactions is essential, for this reason, the experimental data were adjusted to the theoretical models of adsorptions isotherms [55]. Equilibrium sorption data from experiments were described using equations based on isotherms. The equilibrium isotherm parameters offer valuable insights into adsorption mechanisms, the adsorbent’s affinity, and surface characteristics [53]. In this study, various isotherm models, namely Freundlich, Langmuir, and Tempkin, were investigated to assess adsorption equilibrium [56,57,58]. 

The Freundlich isotherm model indicates the presence of heterogeneity in adsorption sites and considers adsorption taking place at sites with varying energy of adsorption. The isotherm is derived from the linear version of the Freundlich expression Equation (7) [54].

(7) $$ln\;q_{e}=ln\;K_{F}+\frac{1}{n_{F}}\;ln\;C_{e}$$

where *q_e_* is the quantity of adsorbed pollutant (mg/g) at equilibrium, *K_F_* is the Freundlich constant (L/g), *C_e_* is the equilibrium concentration of pollutant in solution (mg/L), 1/*n_F_* is the heterogeneity factor.

The Langmuir isotherm model postulates that adsorption takes place at distinct and uniform sites on the adsorbent. This model is widely utilized as an adsorption isotherm, particularly in effectively removing contaminants from aqueous solutions. The Langmuir model´s linearized version is represented by the following Equation (8) [51,57]:

(8) $$\frac{Ce}{q_{e}}=\frac{1}{K_{L}}+\frac{a_{L}}{K_{L}}C_{e}$$

where *q_e_* represents the quantity of adsorbed pollutant (mg/g) at equilibrium, *C_e_* is the equilibrium concentration of the pollutant in the solution (mg/L), *K_L_* (L/g) and *a_L_* (L/mg) are the Langmuir isotherm constants. The parameter q_max_ stands for the utmost adsorption capacity of the adsorbent (mg/g), and its calculation relies on *k_L_*/*a_L_*_;_ The separation factor *R_L_*, determined by Equation (9), is the most important parameter that gives us the Langmuir isotherm. It determines the nature of the adsorption process as unfavorable (*R_L_* > 1), linear (*R_L_* = 1), favorable (0 < *R_L_* < 1) or irreversible (*R_L_* = 0) [59].

(9) $$RL=\frac{1}{1+a_{L}C_{0}}$$

where *C*_0_ is the initial pollutant concentration (mg/L). 

The Tempkin equation establishes that the reduction in adsorption heat as coverage increases follows a linear pattern due to specific interactions between the adsorbate and adsorbent. This adsorption is marked by an even dispersion of bond energies, up to a maximum value [58]. The linear representation of the Tempkin isotherm is given by Equation (10):

(10) $$q_{e}=\frac{RT}{b_{T}}\ln a_{T}+\frac{RT}{b_{T}}\ln C_{e}$$

were *T* represents the absolute temperature in Kelvin; *a_T_* is the constant of the Tempkin isotherm (L/g), *R* is the universal gas constant (8.314 J/mol K), and *b_T_* is the Tempkin constant (kJ/mol) associated with the heat of adsorption.

### 3.7. Polymer Reusability

The reusability of the EPI-β-CDs polymer was evaluated using the same PhACs at 20 mg/L. For that, fifty mL of each PhAC solution was mixed with 1 g of polymer and stirred at 500 rpm for 1 h. Subsequently, the polymer underwent a 10 min separation, and the residual concentration of PhACs was determined spectrophotometrically, as described earlier. The solution containing PhACs was decanted, and the separated polymer was regenerated using a 50 mL acetate buffer solution, pH 4, at a concentration of 220 mM, over a 30 min period. Following this, the polymer was again separated and reloaded with each PhAC for a new usage cycle, up to ten rounds.

### 3.8. Design of a Pilot-Scale Prototype Cyclodextrin Polymer Adsorption of Pollutants

**Figure 4:**
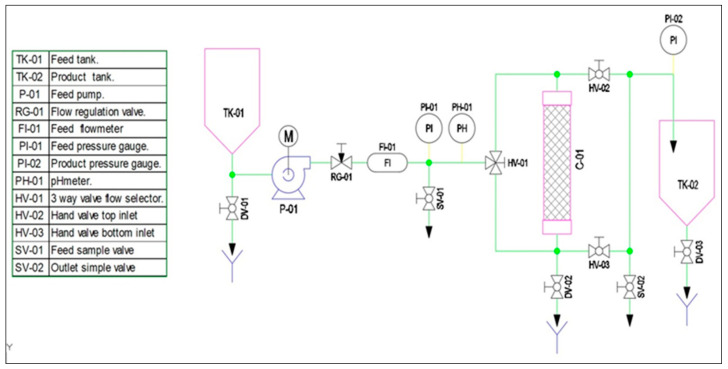
Flow chart of the designed pilot-plant laboratory scale prototype EPI-β-CDs polymer adsorption.

The pilot-plant laboratory scale prototype (PPLSP) was built to ascertain the performances of synthesized EPI-β-CDs polymer in a continuous way (Figure 4) in hereafter studies. 

This PPLSP technological system consisted of two 50 L capacity containers named TK-01 and TK-02; a self-priming polypropylene diaphragm pump P-01, an AISI 316 stainless steel needle valve RG-01; an in-line flow meter FI-01, two pressure gauges, at inlet PI-01 and output PI-02 of the column, a pH meter PH-01, a Ø90 mm adsorption column C-01 to be filled with EPI-β-CDs adsorbent polymer, three-way valve flow selector HV-01 and two hand valves at top HV-02 and bottom HV-03 inlet, and two sample valves for flow feed SV-01 and flow outlet SV-01. 

**Figure 5:**
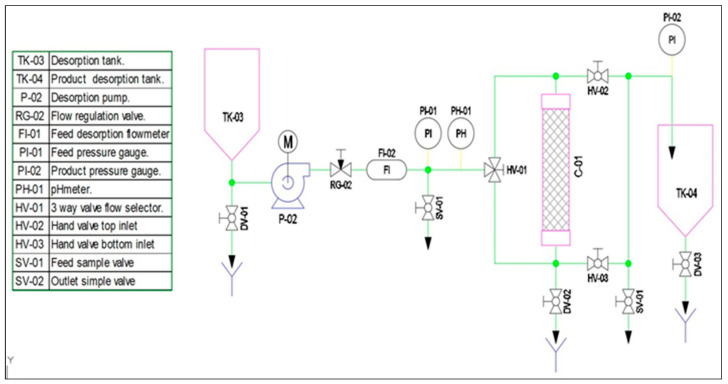
Flow chart of the designed pilot-plant laboratory scale prototype EPI-β-CDs polymer desorption.

Since the prototype will be intended to operate under continuous flows, the adsorbent polymer should be properly regenerated when it loses its capacity, passing through a desorbing solution, to be used repeatedly for the removal of PhACs (Figure 5).

For desorption, the following additional elements: two new 50-L tanks TK-03 and TK-04; a self-priming polypropylene diaphragm pump P-02, an AISI 316 stainless steel needle valve RG-02; and one in-line flow meter FI-02, were included.

## 4. Conclusions

The current study contributes to the efforts in understanding the interaction of furosemide and hydrochlorothiazide with EPI-β-CDs insoluble polymers (particle sizes between 100 and 300 μm), and the design of a lab-scale prototype by predicting continuous adsorption breakthrough from batch assay data.

The experimental data followed the pseudo-second and intraparticle diffusion models. Adsorption occurred onto heterogeneous surfaces according to the three isotherms analyzed. The isotherm models refer to a stronger adsorption and higher capacity of EPI-β-CDs polymer for the furosemide diuretic (1.282 mg/g) compared to hydrochlorothiazide (0.844 mg/g) by considering the number of PhAC mg per g of adsorbent polymer.

The adsorption was exergonic according to the Gibbs free energy results, which indicates the spontaneity of this adsorption process. The polymer demonstrated enhanced reusability, maintaining 90% of its capacity through multiple cycles of loading and desorption for both diuretics. This aligns well with the principles of the circular economy.

The q_max_, density, swelling, particle size, temperature, and EPI-β-CDs polymer water solubility values obtained in batch next to input parameters and process steps, were used to identify and understand each of the stages and equipment involved in building the prototype.

## Figures and Tables

**Table 1:** Adsorption isotherm coefficients obtained for β-CDs-EPI polymer by the Freundlich, Langmuir, and Tempkin models.

Isotherm	Parameter	Furosemide	Hydrochlorothiazide
Freundlich	K_F_ (L/g)	0.044	0.029
	n_F_	0.817	0.737
	R^2^	0.991	0.905
Langmuir	q_max_ (mg/g)	1.282	0.844
	K_L_	0.050	0.038
	a_L_	0.039	0.045
	∆G	−16,919.810	−16,730.651
	R^2^	0.516	0.514
	R_L_	0.838–0.564	0.817–0.527
Tempkin	a_T_	0.525	0.448
	b_T_ (kJ/mol)	6.890	6.79
	R^2^	0.943	0.872

**Table 2:** Values of physical-chemical parameters obtained for β-CDs-EPI polymer for Furosemide and Hydrochlorothiazide by in-batch assay.

Parameter	Value
q_max_ (furosemide) (mg/g)	1.282
q_max_ (hydrochlorothiazide) (mg/g)	0.844
Density (g/cm^3^)	1.06
Swelling	4 ± 1
Particle size (mm)	0.1→0.3
Stability range (pH)	2→11
Temperature range (°C)	5→35
Solubility in H_2_O	Insoluble

**Table 3:** Values of recommended design parameters for a standard adsorption system.

Parameter	Value
Adsorbent Volume (L)	1→3
Column diameter	To define
Adsorbent bed depth (mm)	150→550
Adsorbent expansion (%)	Up to 100
Contact time (min)	1→7.5
Loading flow rate (BV/h)	8→40
Desorbent flow rate (BV/h)	2→5
Desorbent contact time (min)	20→60
Desorbent displacement (BV of water)	2→4
Final rinse (BV service flow rate)	2→10

**Table 4:** Column size design calculations of the prototype.

	Column Size Design Calculations			
Parameters	Ø90 mm		Ø63 mm	
Flow (L/h)	8	40	8	40
Flow rate (m/h)	1.43	7.17	3.10	15.51
Ad volume (L)	1	1	1	1
BV (BV/h)	8	40	8	40
Area (m²)	0.0056	0.0056	0.0026	0.0026
Bed depth (m)	0.18	0.18	0.39	0.39
Expansion (%)	100	100	100	100
Column height (m)	0.36	0.36	0.78	0.78
Contact time (min)	7.5	1.5	7.5	1.5
Flow (L/h)	16	80	16	80
Flow rate (m/h)	2.87	14.33	6.20	31.02
Ad volume (L)	2	2	2	2
BV (BV/h)	8	40	8	40
Area (m²)	0.0056	0.0056	0.0026	0.0026
Bed depth (m)	0.36	0.36	0.78	0.78
Expansion (%)	100	100	100	100
Column height (m)	0.72	0.72	1.55	1.55
Contact time (min)	7.5	1.5	7.5	1.5
Flow (L/h)	24	120	24	120
Flow rate (m/h)	4.30	21.50	9.31	46.54
Ad volume (L)	3	3	3	3
BV (BV/h)	8	40	8	40
Area (m²)	0.0056	0.0056	0.0026	0.0026
Bed depth (m)	0.54	0.54	1.16	1.16
Expansion (%)	100	100	100	100
Column height (m)	1.07	1.07	2.33	2.33
Contact time (min)	7.5	1.5	7.5	1.5

**Table 5:** Theoretical values for flow adsorption cycle estimation of β-CDs-EPI polymer for Furosemide and Hydrochlorothiazide based in batch assay data.

Parameter	Value
PhACs concentration (mg/L)	5→20
Tank volume of PhACs solution (L)	50
Amount PhACs concentration (mg)	250→1000
β-CDs-EPI q_max_ (furosemide) (mg/g)	1.282
β-CDs-EPI q_max_ (hydrochlorothiazide) (mg/g)	0.844
β-CDs-EPI volume (L)	1→3
β-CDs-EPI weight (g/column)	1060→3180
Amount β-CDs-EPI q_max_ (furosemide) (mg)	1358→3846
Amount β-CDs-EPI q_max_ (hydrochlorothiazide) (mg)	894→2683

## Data Availability

Data are contained within the article and Supplementary Materials.

## Supplementary Materials

The following supporting information can be downloaded at: https://www.mdpi.com/article/10.3390/ijms25052829/s1.
