# Supplementary material for: A Comprehensive Strategy for Stepwise Design of a Lab PROTOTYPE for the Removal of Emerging Contaminants in Water Using Cyclodextrin Polymers as Adsorbent Material
Source: Int J Mol Sci. 2024 Feb 29;25(5):2829. doi: 10.3390/ijms25052829 (PMC10931639; doi:10.3390/ijms25052829)
Supplement: Supplementary file 1 [file ijms-25-02829-s001.zip › ijms-2802653-supplementary.pdf]

Supplementary Material for "A comprehensive strategy for stepwise design of a lab prototype for the removal of emerging contaminants in water using cyclodextrin polymers as adsorbent material"

Antonio Tomás Hernández Cegarra [1]; Teresa Gómez-Morte [1]; José Antonio Pellicer [1]; Nuria Vela [2]; María Isabel Rodríguez-López [1], Estrella Núñez-Delicado [1], and José Antonio Gabaldón [1]*

Affiliations:

[1] Molecular Recognition and Encapsulation Research Group (REM), Health Sciences Department, Universidad Católica de Murcia (UCAM), Campus de los Jerónimos 135, E-30107, Guadalupe, Spain

[2] Applied Technology Group to Environmental Health, Universidad Católica de Murcia (UCAM), Campus de los Jerónimos 135, E-30107, Guadalupe, Spain.

*Corresponding author: José Antonio Gabaldón (jagabaldon@ucam.edu)

Supplemental figures

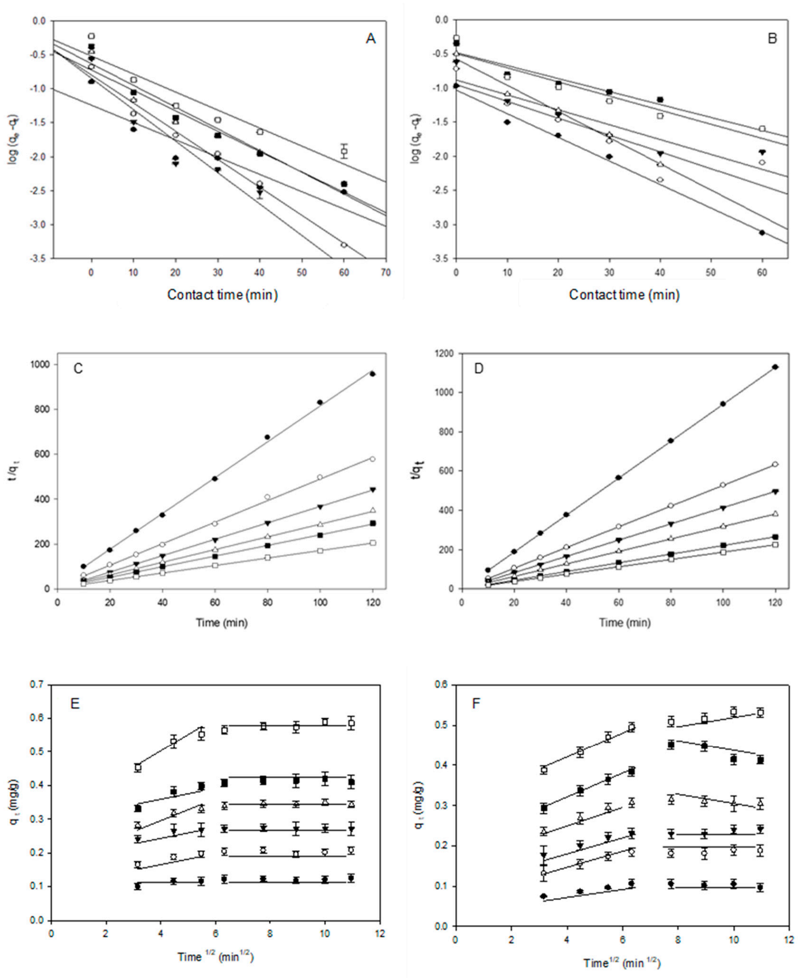

Fig. S1. Kinetic analysis at different concentrations: 5.0 mg/L (●), 7.5 mg/L (○), 10 mg/L (▼), 12.5 mg/L (Δ), 15 mg/L (■), 20 mg/L (□) of pharmaceuticals. Furosemide (A) Pseudo 1st model, (C) Pseudo 2nd model, (E) Intraparticle diffusion mode; Hydrochlorothiazide (B) Pseudo 1st model, (D) Pseudo 2nd model, (F) Intraparticle diffusion mode; N=3.

Commented [g1]: Symbol has been changed properly

Supplemental figures

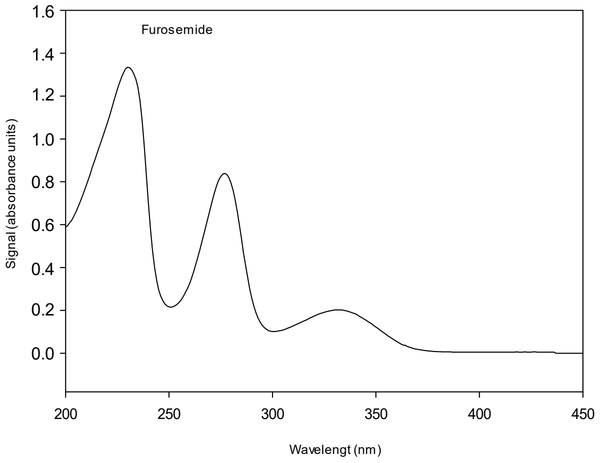

Fig. S2. Absorption spectra of furosemide

.

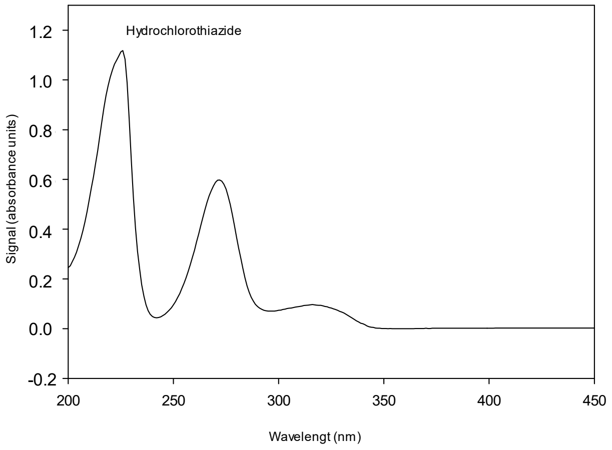

Fig. S3. Absorption spectra of hydrochlorothiazide

Supplemental tables

Table S1. β-CD-EPI polymer kinetic parameters (pseudo 1st, pseudo 2nd and intraparticle diffusion models).

PFOM		β-CDs-EPI polymer and Furosemide		
Co (mg/L)	qe_{exp}	qe_{cal}	K_1 (min-1)	R^2
5	0.126	0.057	0.059	0.834
7.5	0.208	0.158	0.095	0.987
10	0.275	0.142	0.106	0.895
12.5	0.350	0.187	0.069	0.936
15	0.419	0.232	0.073	0.946
20	0.588	0.301	0.061	0.896
**PSOM**		**β-CDs-EPI polymer and Furosemide**		
Co (mg/L)	qe_{exp}	qe_{cal}	K_2 (g/mg min)	R^2
5	0.126	0.126	3.691	0.998
7.5	0.208	0.208	2.380	0.998
10	0.275	0.273	6.500	0.999
12.5	0.350	0.353	1.432	0.999
15	0.419	0.422	1.300	0.999
20	0.588	0.601	0.622	0.999
**IDM**		**β-CDs-EPI polymer and Furosemide**		
Co (mg/L)	qe_{exp}	qe_{cal}	K_i (mg/g min 1/2)	R^2
5	0.126	0.102	0.002	0.602
7.5	0.208	0.168	0.004	0.576
10	0.275	0.249	0.003	0.470
12.5	0.350	0.283	0.007	0.701
15	0.419	0.336	0.009	0.639
20	0.588	0.455	0.014	0.715
**PFOM**		**β-CDs-EPI polymer and Hydrochlorothiziade**		
Co (mg/L)	qe_{exp}	qe_{cal}	K_1 (min-1)	R^2
5	0.106	0.093	0.080	0.991
7.5	0.190	0.803	0.057	0.805
10	0.242	0.131	0.050	0.834
12.5	0.315	0.264	0.088	0.984
15	0.452	0.328	0.044	0.889
20	0.534	0.318	0.048	0.903
**PSOM**		**β-CDs-EPI polymer and Hydrochlorothiziade**		

Co (mg/L)	qe_{exp}	qe_{cal}	K_2 $^{(g/mg\ min)}$	R^2
5	0.106	0.106	7.322	0.992
7.5	0.190	0.190	1.187	0.999
10	0.242	0.242	0.950	0.999
12.5	0.315	0.315	1.593	0.999
15	0.452	0.452	0.513	0.992
20	0.534	0.534	0.350	0.999
**IDM**	**β-CDs-EPI polymer and Hydrochlorothiziade**			
Co (mg/L)	qe_{exp}	qe_{cal}	K_i $^{(mg/g\ min\ 1/2)}$	R^2
5	0.106	0.077	0.003	0.471
7.5	0.190	0.129	0.006	0.716
10	0.242	0.171	0.007	0.792
12.5	0.315	0.237	0.008	0.595
15	0.452	0.270	0.017	0.685
20	0.534	0.359	0.018	0.885

Table S2. On/off positions of the valves when working co-current flow.

Up-Down Flow		
	Drive	
TAG	On	Off
HV01	X	
HV02		X
HV03	X	
DV01		X
DV02		X
DV03		X
SV01		X
SV02		X
SV03		X
P01	X	
RG-01	X	

Table S3. On/off positions of the valves when working counter-current flow.

Down-Up Flow		
	Drive	
TAG	On	Off
HV01	X	
HV02	X	
HV03		X
DV01		X
DV02		X
DV03		X
SV01		X
SV02		X
SV03		X
P01	X	
RG-01	X	

Table S4. On/off positions of the valves to carry out the desorption process.

Desorption process		
	Drive	
TAG	On	Off
HV01	X	
HV02	X	
HV03		X
DV01		X
DV02		X
DV03	X	
SV01		X
SV02		X
SV03		X
P02	X	
RG-01	X	

Table S5. Design calculations for an additional smaller diameter column Ø63 mm not exceeding the necessary 1,200 mm height.

Column size design calculations		
Ø63 mm		
Flow (L/h)	4.8	24
Flow rate (m/h)	1.9	9.4
Adsorbent volume (L)	0.6	0.6
BV (BV/h)	8	40
Area (m^2)	0.0026	0.0026
Bed depth (m)	0.24	0.24
Expansion (%)	100%	100%
Column height (m)	0.47	0.47
Contact time (min)	7.5	1.5
Flow (L/h)	8	40
Flow rate (m/h)	3.1	15.7
Adsorbent volume (L)	1	1
BV (BV/h)	8	40
Area (m^2)	0.0026	0.0026
Bed depth (m)	0.39	0.39
Expansion (%)	100%	100%
Column height (m)	0.78	0.78
Contact time (min)	7.5	1.5
Flow (L/h)	11,2	56
Flow rate (m/h)	4.4	21.9
Adsorbent volume (L)	1.4	1.4
BV (BV/h)	8	40
Area (m^2)	0.0026	0.0026
Bed depth (m)	0,55	0,55
Expansion (%)	100%	100%
Column height (m)	1.10	1.10
Contact time (min)	7.5	1.5
